# ENCoRE: an efficient software for CRISPR screens identifies new players in extrinsic apoptosis

**DOI:** 10.1186/s12864-017-4285-2

**Published:** 2017-11-25

**Authors:** Dietrich Trümbach, Susanne Pfeiffer, Manuel Poppe, Hagen Scherb, Sebastian Doll, Wolfgang Wurst, Joel A. Schick

**Affiliations:** 1 Institute of Developmental Genetics, Helmholtz Zentrum Munich, Ingolstädter Landstraße 1, 85764 Neuherberg, Germany; 2 Institute of Molecular Toxicology and Pharmacology, Helmholtz Zentrum Munich, Ingolstädter Landstraße 1, 85764 Neuherberg, Germany; 3 Institute of Computational Biology, Helmholtz Zentrum Munich, Ingolstädter Landstraße 1, 85764 Neuherberg, Germany; 4 Technische Universität München-Weihenstephan, Chair of Developmental Genetics c/o Helmholtz Zentrum München, Ingolstädter Landstr. 1, 85764 Neuherberg/Munich, Germany; 5 German Center for Neurodegenerative Diseases (DZNE) Site Munich, Feodor-Lynen-Str. 17, 81377 Munich, Germany; 6 Munich Cluster for Systems Neurology (SyNergy), Feodor-Lynen-Str. 17, 81377 Munich, Germany

**Keywords:** CRISPR, Cas9, Screen, Software, TNFalpha, Apoptosis

## Abstract

**Background:**

As CRISPR/Cas9 mediated screens with pooled guide libraries in somatic cells become increasingly established, an unmet need for rapid and accurate companion informatics tools has emerged. We have developed a lightweight and efficient software to easily manipulate large raw next generation sequencing datasets derived from such screens into informative relational context with graphical support. The advantages of the software entitled ENCoRE (**E**asy **N**GS-to-Gene **C**RISPR **RE**sults) include a simple graphical workflow, platform independence, local and fast multithreaded processing, data pre-processing and gene mapping with custom library import.

**Results:**

We demonstrate the capabilities of ENCoRE to interrogate results from a pooled CRISPR cellular viability screen following Tumor Necrosis Factor-alpha challenge. The results not only identified stereotypical players in extrinsic apoptotic signaling but two as yet uncharacterized members of the extrinsic apoptotic cascade, *Smg7* and *Ces2a*. We further validated and characterized cell lines containing mutations in these genes against a panel of cell death stimuli and involvement in p53 signaling.

**Conclusions:**

In summary, this software enables bench scientists with sensitive data or without access to informatic cores to rapidly interpret results from large scale experiments resulting from pooled CRISPR/Cas9 library screens.

**Electronic supplementary material:**

The online version of this article (10.1186/s12864-017-4285-2) contains supplementary material, which is available to authorized users.

## Background

CRISPR/Cas9 technology has enabled rapid genetic mutation in mammalian cells and the ability to conduct genome-wide screens using tailored lentiviral libraries [1–9]. The system consists of a sgRNA (single guide RNA) that directs the paradigm Cas9 nuclease from *Streptococcus pyogenes* to bind and cleave genomic DNA strands at the location corresponding to the guide sequence [10]. The resulting double strand cleavage triggers cellular repair mechanisms including non-homologous end-joining (NHEJ), which in the imperfect sense generates insertions or deletions (indel) mutations and associated nonsense transcripts. This technology makes pooled sgRNA libraries a powerful tool to perform genome-wide screens for both dominant and recessive genes. The resulting unique cellular subtypes generated are typically screened in pools for identifying hallmarks, i.e., survival or fluorescence reporter activation and guide distributions are then identified by next generation sequencing (NGS). Newer Cas9 technologies have also been developed to up- or down-regulate gene expression level, setting the stage for screens involving more subtle changes leading to desired phenotypic outputs [1, 11–13]. In addition, non-coding DNA is also rapidly becoming a popular target for such screens [14, 15].

In contrast to other methods such as near-haploid genetic screens where viral insertion sites must be determined by sequencing [16], the pooled library lentiviral-derived sgRNA sequences are pre-determined. Thus, guide sequences amplified from the genomic DNA of selected cells serve as a simple proxy to identify active genes or pathways in selected cells. However, as with other large scale technologies, the results of such screens are often primary next generation sequencing files, which unfortunately because of their size and format are unwieldy depots of information for bench scientists. Moreover, data processing, particularly from raw sequences to sgRNAs, often requires processing from different sources. An optimal solution is therefore to combine workflow steps into a single package. Here, we sought to create a complete CRISPR analysis software package with a simplified graphical workflow that can enable bench scientists to keep pace with large-scale data generation in by quickly processing NGS sequence files and generating graphical outputs with statistical representation. We demonstrate the power of ENCoRE to rapidly deliver results in a cell survival screen using Tumor Necrosis Factor-alpha (TNFa) challenge, which identified known members as well as two genes not yet implicated in the extrinsic apoptosis pathway, *Smg7* and *Ces2a*. Further characterization of cell lines containing mutations in these genes shows that they lie predominantly in the extrinsic apoptosis pathway and are not generally protective against cell death stimuli. Both cell lines show an increase in p53 protein, consistent with an abrogated p53 pro-apoptotic signaling cascade, with *Smg7 −/−* cells showing the most substantial increase following TNFa treatment.

## Implementation

### CRISPR screening

Immortalized mouse fibroblasts were infected with ecotropic lentivirus [4] particles containing the Cas9-Blasticidin construct essentially as described in [17]. The genome-wide mouse lentiviral CRISPR gRNA library was a gift from Kosuke Yusa (Addgene #50947). Cells were treated with 10 μg/mL blasticidin (Sigma) for five days and resistant cells were used for a second infection with ecotropic lentivirus particles containing sgRNA expressing library (pKLV-U6gRNA-PGKpuro2ABFP) at an MOI of 0.3. Following infection, cells were sorted using Blue Fluorescent Protein (BFP) as a marker. The BFP-positive cell population was seeded onto a 15 cm dish at low density (100–500 cells per 15 cm plate) and treated with TNFa (Life Technologies, 20 ng/mL) or control for 11 days. Following this, genomic DNA was isolated from the control and selected pools and samples prepared for sequencing on a Ion Torrent P1 chip (PrimBio Research Institute, Exton, PA), see [Additional file 1] for primer sequences. Using the FASTQ Filter Module (FFM, below) ENCoRE identified all single reads contained in the FASTQ file. Sequencing reads were then trimmed directly after the search sequence ‘gaaacaccg’ at 3′ ends consisting of 19 bp also in the FFM module. The residual 19 bp sequence corresponded with the CRISPR guide RNA sequence in the CRISPR Report Module (CRM). The dataset was then compressed and repeated guides were counted and integrated into unique guide sequences. The CRM module was used to compare the counts of individual guides from selected libraries with the unselected library and the total starting library. Candidate genes were chosen for subsequent validation based on individual guide results and literature survey.

### ENCoRE and statistical calculations

ENCoRE was custom written in Java programming language in the Eclipse programming environment (released June 2015). Binaries, source code, and classes can be downloaded from [18]. For usage and a detailed walkthrough see the ENCoRE Quick Guide [Additional file 2]. The method implemented in the CRM of ENCoRE works as follows:

1. Filtered reads from experimental and the control FASTQ files filtered by FFM are counted and assigned to gene names or identifier by mapping the guide sequences and identifiers from the reference file in the CRM. For the TNFa screen, the reference file from [4] was used. Experimental and control replicate FASTQ files are imported in the same way. A data matrix is generated by a HashMap in Java where the gene names/identifiers represent the rows and the read counts of different experiment and control guides as the columns.
2. Missing values are set to one in the data matrix. Counts of one are afterwards replaced by the median of all sgRNAs counts in the experiment and separately in the control for each gene. This procedure is denoted as median imputation.
3. Total normalization is applied by dividing each read count (after median imputation) for a given library through the sum of all counts (after imputation).
4. Based on the normalized read counts, the log_2_-ratios between corresponding experiment and control guides are calculated for each gene (e.g., five different log_2_-ratios in case of the TNFa dataset). With help of these log_2_-ratios the mean, the standard deviation and the t-score is computed for each gene. Finally, from the t-score the *p*-value is determined from the normal distribution by the Java method “cumulativeProbability” of the Apache Commons Mathematics Library. The described procedure is equivalent to paired comparisons in the paired t-test.
5. The *p*-values are corrected for multiple testing with help of the method according to [19] as well as to Bonferroni.
6. The data matrix of imputated and original counts as well as *P* value, false discovery rate and log_2_[fold change] is automatically exported by ENCoRE in comma separated values (CSV) format after generating the volcano plot.

### ENCoRE comparisons

For the TNFa screen and Koike-Yusa, et al., [4] data an exact negative binomial test from the Bioconducter package edgeR [20] was applied to the original counts obtained after filtering the corresponding FASTQ files by FFM of ENCoRE. For Bassett, et al., [21] the prefiltered FASTQ files (GSE67339 series from GEO database) were used for the analysis by edgeR. We followed the recommendations of the edgeR manual and performed a normalization with help of the function calcNormFactors(). Further, the common and tag-wise dispersion was estimated using the functions estimateCommonDisp() and estimateTagwiseDisp(), respectively. To obtain positively selected genes the resulting lists by the R function topTags() were filtered for positive fold changes (logFC) and subsequently ordered by increasing *p*-values. Filtered FASTQ files of each study were additionally analyzed with standard settings in MAGeCK [22] using the command “mageck count” and subsequently “mageck test” to derive ranked lists of positively selected genes. Venn diagrams [23] were generated by combining the top 20 ranked genes of the ENCoRE, MAGeCK and edgeR analyses for the three data sets.

### Cell line mutagenesis

Individual gene null mutations in mouse fibroblasts were performed using guide sequences (see [Additional file 1] for guide sequences) cloned into the pKLV-U6gRNA(BbsI)-PGKpuro2aBFP vector (a gift from Kosuke Yusa (Addgene plasmid # 50946)), ecotropic virus was generated and cells infected as above. BFP(+) colonies were then screened for mutations using PCR primers designed outside the guide sequence. PCR products were cloned using the Zero Blunt TOPO PCR Cloning Kit (ThermoFisher) and Sanger sequenced with recommended primers.

### Cellular assays

Mutant and control (parental) cell lines were plated the day before treatment at a density of 2000 cells/well on 96 well plates and were treated the following day with following factors: TNFa (LifeTechnologies, PMC3014, 100 μg/ml) for 24 h, Erastin (Sigma, E7781, 10 mM) for 24 h, Doxorubicin (edom, D2975000, 10 mM) for 24 h, Vinblastine (Cayman, 11,762, 10 mg/ml) for 24 h, Staurosporine (Sigma, S5921, 10 mM) for 24 h, Taxol (Sigma, T7402, 1 mM) for 72 h, Deoxycholic acid (DCA, Sigma, D2510, 500 mM) for 24 h, or 5-Fluorouracil, (5-FU, Sigma, F6627, 50 mM) for 72 h. Cycloheximide (CHX, Merck, 239,764–1, 100 mg/ml) and zVAD (Enzo, BML-P416–0001, 10 mM) were added to culture medium where indicated. Following treatment, media was changed to viability reagent AquaBluer (MultiTarget) containing according to manufacturer instructions and fluorescence change was measured at 540 nM excitation/590 nM emission. Cells were treated in three separate experiments and analyzed three days after the first treatment unless otherwise specified. Selected antibodies were purchased for p65 (Santa Cruz), Caspase-8 (Cell Signaling), Smg7 (Biomol), Ces2a (LSBio), PAN-actin (Cell Signaling), and p53 (Cell Signaling). The translocation assays were performed on an Operetta High-Content System (PerkinElmer) using DAPI to identify primary objects and Cy3 antibody (Dianova) label intensity and/or localization. Induction was performed with TNFa (20 ng/mL) for 5 h. The NF-κB induction assay was carried out using pTK Renilla control and p6xNFkB Luciferase (Promega) plasmids and output measured by Dual-Glo (Promega). Induction was performed with TNFa (20 ng/mL) for 2 h and normalized to the Renilla control. The Western assay was performed with 16 h induction with TNFa (20 ng/mL). Statistical significance was determined using GraphPad Prism software and two-tailed t-test or two-way, repeated-measures ANOVA with a Bonferroni post-test as indicated.

## Results

### ENCoRE software for NGS processing

**Fig. 1:**
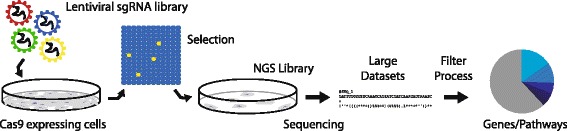
An overview of a typical CRISPR screen. Lentiviral particles containing pooled guide sequences are added to cells expressing Cas9 nuclease. Following selection and amplification, guides are deep sequenced from genomic DNA, filtered, and correlated to genes or chromosomal position. Statistical analysis and pathway deconvolution complete the initial assessment after which candidates are individually validated

A typical CRISPR/Cas9 library screening workflow involves three basic steps: introduction of the pooled sgRNA library into cells stably expressing the Cas9 nuclease, followed by a phenotypic selection and finally sequencing of the lentiviral guide sequences from the genomic DNA of selected cells (Fig. 1).

The final output is generally PCR-amplified sgRNA sequence performed on isolated DNA with NGS technology specific adapters. As the guide sequences in the selected cells are presumed to contain mutations in the corresponding gene, sequencing of sgRNAs from the cells provides a simple proxy to identify candidate genes in the selected process. Most libraries contain several unique guide sequences per gene to increase the likelihood of mutation and statistical reliability. Following sequencing, FASTQ data files generally comprising several gigabytes of data are produced.

**Fig. 2:**
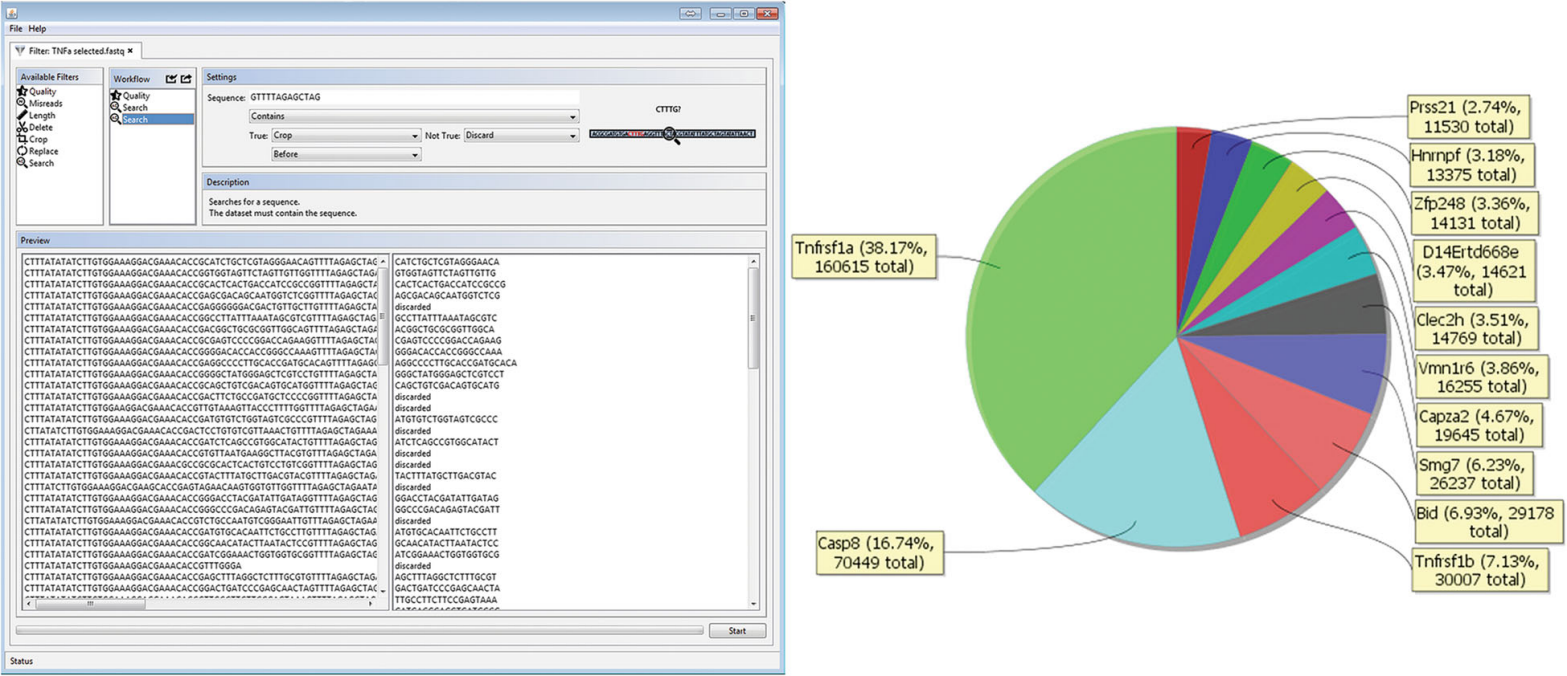
The User Interface of ENCoRE. ENCoRE comprises two modules: the FASTQ Filter Module (FFM) and the CRISPR Report Module (CRM). The FFM (left) is used to curate primary FASTQ sequencing files and to remove extraneous sequences, leaving CRISPR guide sequences intact. Workflows can be saved to maintain processing fidelity between multiple files. The CRM (right) compares guides to a reference file to generate designations (for example, gene, chromosomal location) according to user. The resulting absolute guide representation is displayed in form of a configurable pie chart, with the option to aggregate guides directed against a given target. Illustrative results are shown here for the TNFa challenge described below

In order to retrieve valid sequences the data files must be manipulated and filtered prior to matching against a reference library. Several programs use a console-based input and are therefore cumbersome for inexperienced users. ENCoRE addresses this problem by serving up a slim and convenient graphical user interface (GUI), mimicking the look and feel of the current operating system (Fig. 2). Two separate modules are embedded: a FASTQ Filter module (FFM) for sequence manipulation and quality control, and a CRISPR Report module (CRM) that compares the filtered sequences to a reference file and displays the results.

Filtering and curating large FASTQ datasets represents the core preprocessing module of ENCoRE. The module provides extensive filters for sequences like crop, cut, replace, search and quality analysis. These filters can be arranged in a pipeline to sequentially alter every dataset in large FASTQ files. To facilitate the assembly of a filter workflow, ENCoRE provides the user with a live preview of the produced output sequences for every workflow step (Fig. 2). To ensure consistent filtering workstreams for multiple FASTQ files, workflow settings can be exported for use on other datasets. Possibly the most useful function is a generalized string search that allows for rapid identification and cropping of library anchor and priming sequences. Thus, primer and library tag sequences can be efficiently identified for removal while barcodes can be recognized to deconvolute multiplexed experiments. The final processing is done using a multithreaded worker-consumer pattern that scales to the local machine and fully exploits its processing power. This way it is possible to process 15 gigabyte data files within a few minutes on a typical desktop. The program runs fully localized as required for sensitive data (i.e., involving human subjects or intellectual property) without external databases. The output is saved to a filtered FASTQ file for further use, e.g., quality control or processing in the report module.

After appropriate filters are applied, the CRISPR Report Module is able to match the resulting sgRNA ‘tags’ to a corresponding reference file for a given sgRNA library. These files unfortunately lack a standardized format. However, the CRM has the power to adapt to any present reference file prepared in a (CSV) format including headers. In this way, the CRM is compatible with virtually all present sgRNA libraries but also with future CRISPR iterations using tailored sgRNA libraries or ones not based on *Streptococcus pyogenes* Cas9 [24–26]. To display numerical proportion of genes and the ratio of successful sgRNAs, a display chart can be generated using the guide information of a filtered FASTQ file. The CRM enables the user to show basic information with a pie chart representing all sequenced reads and their corresponding absolute number of isolated guide sequences (Fig. 2). Inputs derive from reference file column headers and allow for visualization by all criteria in the reference library (i.e., guide sequence, gene name (aggregated or unaggregated), chromosomal position). A slider allows for selective data zooming and automatically emphasizes enriched hits with relevant count information. The import and processing are performed in a separate thread, keeping the GUI active and enabling the user to interact with the chart even though data is still processed. An export function allows for saving the read counts linked with the corresponding guide in a CSV file for further analysis.

**Fig. 3:**
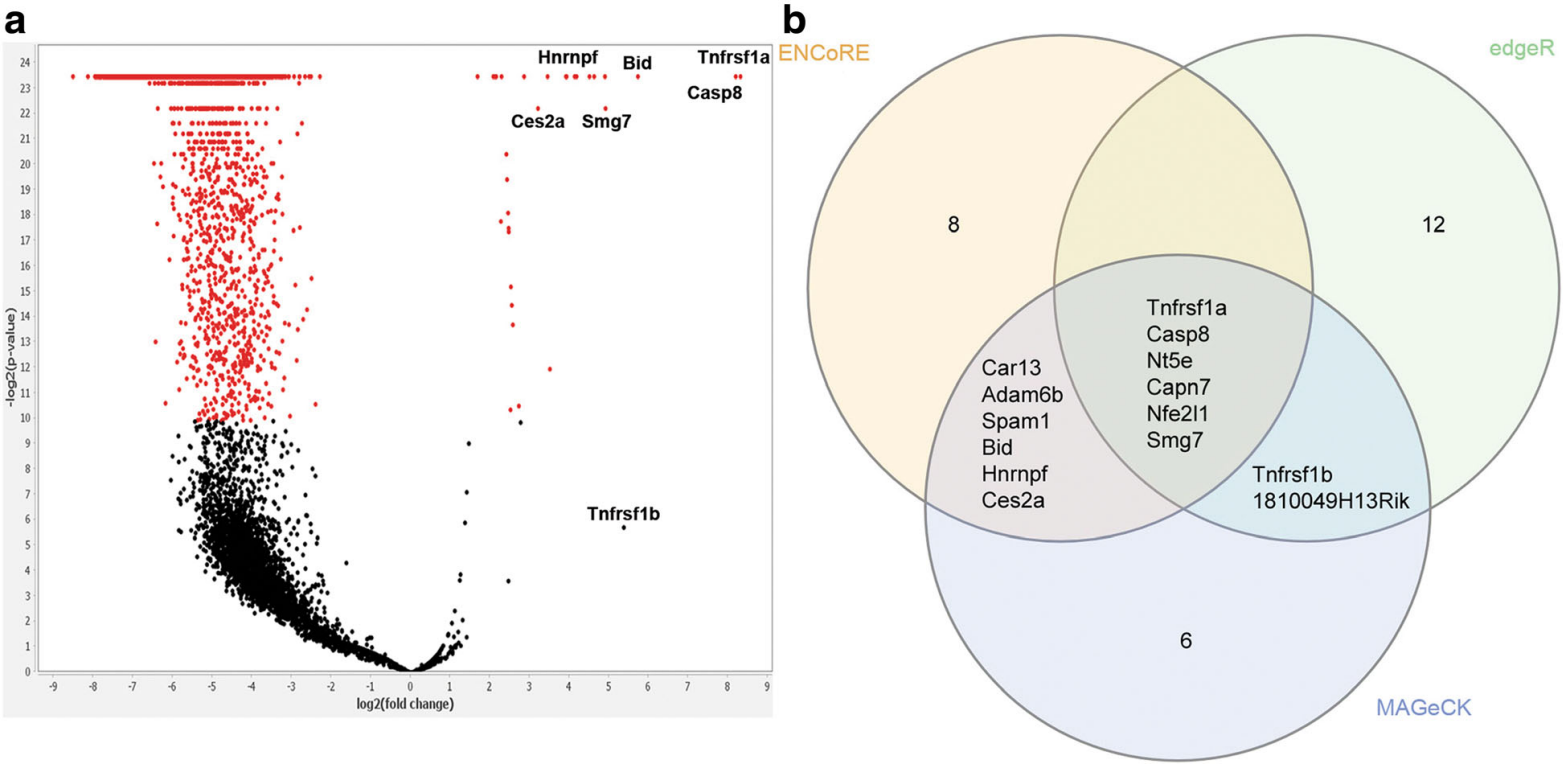
Extrinsic apoptosis pathway members identified by ENCoRE following TNFa challenge. **a** ENCoRE’s CRISPR Report Module indexing method produces a volcano plot that combines fold change with *p*-values. Higher scoring genes appear farther right and near the top. Known apoptosis genes and new candidate genes chosen for validation are highlighted. **b** Comparison of top 20 ranked genes identified by ENCoRE, MAGeCK and edgeR following TNFa challenge shows substantial overlap between top hits

The relative change in sgRNA frequency (sgRNA_experimental_/sgRNA_control_) found for a given gene corresponds to its likelihood of being involved in the tested process [4]. Therefore, CRM combines the fold change for all guides corresponding to a given gene and compares those to an unselected control sample. To compare experimental frequencies (counts) with control counts specific for several guides, it is convenient to assume negative binomially distributed counts and to carry out a generalized regression, which estimates and tests the main treatment effects adjusted for the dummy variables representing the guides. This is essentially equivalent to a paired comparison, similar to the well-known paired t-test. However, a straightforward alternative and approximate solution to this is a normal z-test directly applied to the means of the log_2_[fold change] of aggregated guides per gene. Comparison of this method to analysis software MAGeCK [22] and edgeR [20] led to comparable overlaps of top ranked sgRNAs for published datasets, and particularly for data from this study (Additional file 3, Fig. 3b). To compensate for missing or underperforming guides, either due to incomplete NGS data or failure for the guide to generate a phenotypic change, a median imputation strategy was defined prior to the z-test. This means that missing counts are replaced by the median of the counts. The median imputation is less susceptible to outlier impact as compared to mean imputation. The CRM finally displays a simple graphical output in form of a volcano plot of −log_2_[*p*-value] where *p*-values are derived from a z-test versus the log_2_[fold change]. The threshold for a Bonferroni correction considering about 20,000 genes (i.e. aggregated guides) can be calculated by 0.05/20000 = 0.0000025. In order to highlight also guides that show clear significance a Bonferroni threshold was chosen by red dots in the volcano plot. This graph allows simple visualization of genes with increased relevance and is useful when relative sgRNA increases are small, particularly under imperfect selection conditions such as for TNFa (Fig. 3; below). This allows the user to quickly examine expected results and define candidates for further tests also with the individual aggregated fraction of total guides. Mouseover, zoom and capture are also possible for data presentation. Finally, after processing and importation, the identifier data with statistical output are stored in a CSV file for further analysis in other software. ENCoRE supports multiple group inputs, enabling studies with repeated measures to be analyzed and exported as with single experimental studies.

### Extrinsic apoptosis pathway validation

In order to demonstrate the power of ENCoRE, we conducted a CRISPR screen using a library of ~88,000 sgRNAs [4] in mouse fibroblasts to identify members of the extrinsic apoptosis pathway induced by TNFa, a well-characterized extrinsic apoptosis inducing agent. In our hands, TNFa incompletely induces apoptosis in a cell population, thus substantial background sgRNAs would be expected to confound results. Indeed, we observed only approximately 60% cell death at the highest concentration tested (40 ng/mL) following treatment, shown in [Additional file 4]. Therefore, we sought to test if meaningful results could be inferred from a screen conducted under imperfect selection conditions.

Using this methodology, we sought to validate ENCoRE with the identification of canonical members of the extrinsic apoptosis pathway. Immortalized mouse fibroblasts expressing stable Cas9 [27] were infected with a lentiviral genome-wide CRISPR guide library directed against coding sequences with up to five unique guides per gene [4]. After TNFa (20 ng/mL) treatment was applied, mutant cell pools were harvested and genomic DNA was extracted. Genomic DNA from unselected cells served as a control population as well as pure plasmid preparation of the sgRNA library. Primers were designed to bind to the lentiviral vector backbone and amplify a small (194 bp) DNA fragment including the variable guide region to generate a library suitable for next generation sequencing. ENCoRE was then used to process the resulting library file and extract gene information as described below.

ENCoRE uses a classical method to represent significance versus the frequency of gene mutations (log_2_[*p*-value] versus –log_2_[fold change]; Fig. 3a). This metric allows for simple classification of productive sgRNA hits and produces a traditional volcano plot result by highlighting *p*-values that are significant (*Padj* < 0.05) after Bonferroni correction. ENCoRE successfully identified canonical members within the top genes, including *Tnfrsf1a* (0.20% of all sgRNA sequences, *Padj* < 0.0000001), *Casp8* (0.91% guides, *Padj* < 0.0000001), *Bid* (0.17% guides, *Padj* = 0.00000006) and *Tnfrsf1b* (0.54% guides, *Padj* = 0.02277213) (Fig. 3a). Comparison to MAGeCK and edgeR showed substantial overlap among the top 20 discovered genes (12 and 6 genes, respectively) supporting the role of these genes in cell death signaling and ENCoRE methodology (Fig. 3b). The discovery of other apoptosis-promoting factors was not unexpected, for example the heterogeneous nuclear ribonucleoprotein F protein (*Hnrnpf*; *Padj* = 0.0000279) that regulates splicing of the B-cell lymphoma/leukemia-2 (Bcl-2)-related family member *Bcl-x* (RNA) [28]. However, the data also revealed a surprising result for two new genes previously uncharacterized in extrinsic apoptosis: the nonsense mediated mRNA decay factor, suppressor with morphological defects in genitalia 7, (*Smg7*; 0.33% guides, *Padj* = 0.00356165), and the carboxylesterase *Ces2a* (0.11% guides, *Padj* = 0.0041534), which should not to be confused with the ces-2 transcription factor from *C. elegans* also involved in cell death. We directed our attention to these genes in further experiments to determine their roles in regulating cell death signaling.

**Fig. 4:**
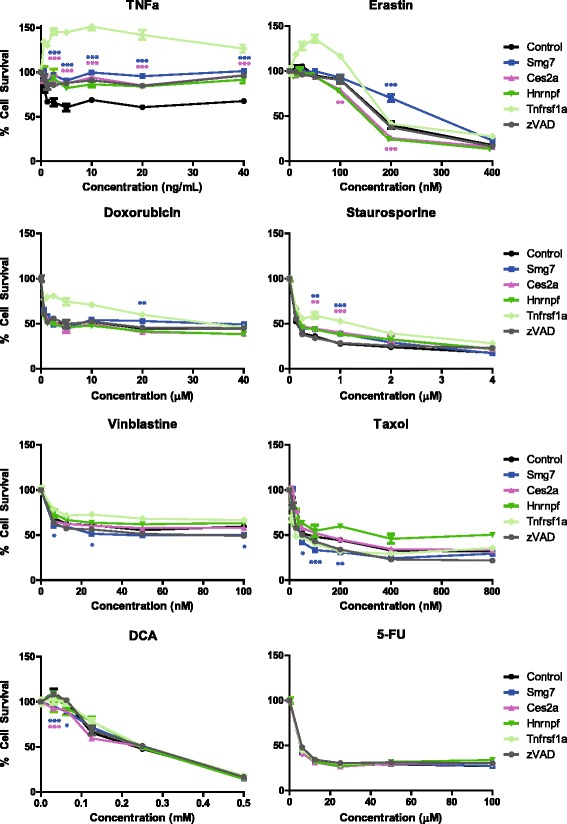
Challenge of mutant cell lines to different cell death stimuli. The control (parental) cell line as well as individually engineered null mutant cell lines (*Smg7, Ces2a, Hnrnpf, Tnfrsf1a*) were compared for resistance to a panel of cell death inducing agents. Two-way, repeated-measures ANOVA with a Bonferroni post test showed *Smg7* and *Ces2a* mutant cell lines to be statistically different from control cells at different doses (colored asterisks: above, more resistant; below, more sensitive. Statistics of other cell lines not shown). *P < .05. **P < .01. ***P < .001. Z-Val-Ala-Asp-fluoromethylketone (zVAD) was added to control cells to demonstrate caspase-dependent cell death. Abbreviations: Deoxycholic acid (DCA); 5-Fluorouracil, (5-FU)

To directly test whether *Smg7* and *Ces2a* contribute to extrinsic apoptosis, we generated independent indel null mutations each of the above genes using CRISPR, shown in [Additional file 5], in parallel with mutations in *Tnfrsf1a* and *Hnrnpf* as positive controls. Following characterization of clones, we tested individual cell lines containing mutations in both alleles for resistance to TNFa challenge (Fig. 4). Mutations in all cell lines showed significant differences (*P* < 0.001, two-way, repeated measures ANOVA) from the parental cell line upon exposure to a range of TNFa concentrations with *Tnfrsf1a −/−* cells showing even an increase in cell number, consistent with a separate role for TNFa in survival and proliferation when apoptosis is not activated [29]. *Smg7 −/−* and *Ces2a −/−* cells also displayed robust protection compared to other cell lines at all concentrations tested. The control pan-caspase inhibitor z-VAD-FMK (zVAD) showed significant protection against TNFa challenge, demonstrating that the four discovered genes are bona fide factors that promote caspase-dependent extrinsic apoptosis.

Next, to test whether the *Smg7* and *Ces2a* specifically function in regulation of extrinsic apoptosis we induced cell death by other modalities. First, we tested for resistance to the ferroptosis inducer, Erastin [30]. *Smg7 −/−* and *Tnfrsf1a −/−* cells showed significant protection against Erastin (*P* < 0.001) at concentrations up to 200 nM whereas *Ces2a −/−* and *Hnrnpf −/−* were more sensitive than control cells at this concentration. Minor protection against doxorubicin induced cell death was also observed in *Smg7 −/−* cells at 20 μM and *Smg7 −/−* and *Ces2a −/−* cells were both found to be partially resistant to staurosporine at low doses. However, *Smg7 −/−* cells were more sensitive than control cells to vinblastine and Taxol, and both cell lines were sensitive to low doses of Deoxycholic acid (DCA). *Hnrnpf −/−* cells showed significant resistance at several concentrations to Taxol (200, 800 nM; *P* < 0.001) likely resulting from protein interaction with HNRNPA2, a prognosticator for Taxol resistance in ovarian cells [31, 32]. Cumulatively, these results show that mutation of either *Smg7* or *Ces2a* robustly protects against extrinsic apoptosis induced by TNFa challenge. In addition, mutation of *Smg7* can also partially protect against cell death induced by ferroptosis and doxorubicin, whereas both *Smg7* and *Ces2a* mutant cell lines showed partial resistance against staurosporine at low concentrations but not against other drugs used in oncology.

**Fig. 5:**
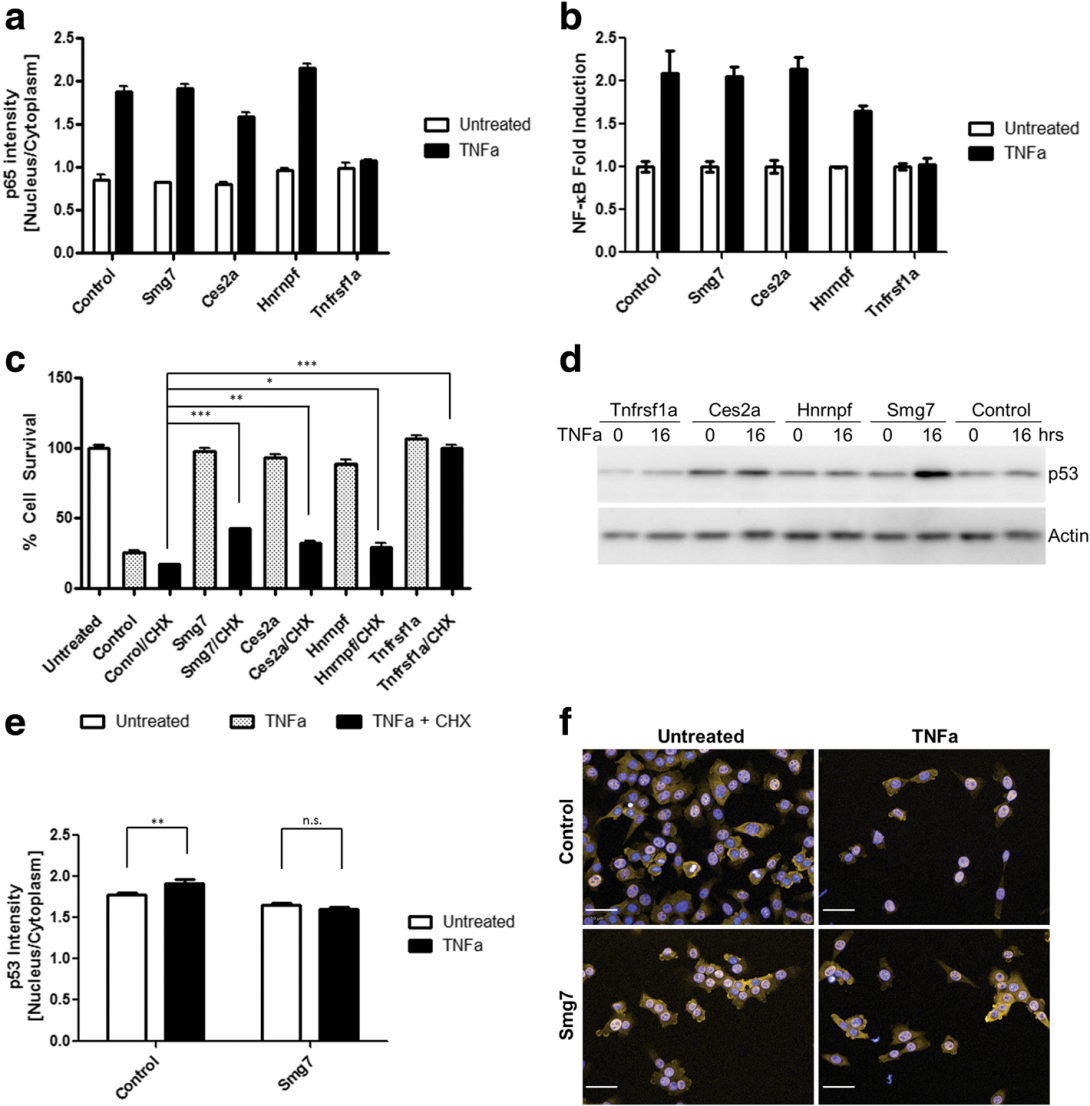
Characterization of TNFa-induced apoptosis in *Smg7 −/−*, *Ces2a −/−* and control cell lines. **a** The NF-κB responsive p65 protein was detected by high-content microscopy and the ratio of nuclear:cytosolic forms was compared for the mutant cell lines in untreated and TNFa treated conditions. **b** The NF-κB transcriptional response was measured by luciferase assay and fold induction calculated relative to a transfection control. **c** *Smg7 −/−*, *Ces2a −/−, Hnrnpf −/−* cell lines can be resensitized to TNFa by addition of cycloheximide (CHX) but are significantly less sensitive than the control. *Trnfrsf1a −/−* cells cannot be resensitized. **d** Cell death proteins were detected by Western blot at 0 and 16 h of TNFa treatment. Basal levels of p53 are upregulated in *Ces2a −/−* and in *Smg7 −/−* cells following TNFa addition. **e, f** Control cells show a significant increase in nuclear (DAPI-stained) fraction of p53 (red) following TNFa treatment while *Smg7 −/−* cells show similar levels. *P < .05. **P < .01. ***P < .001. n.s. not significant. Scale bar is 50 μm

Next, we tested if mutations in *Smg7* and *Ces2a* activate the protective NF-κB transcriptional Complex I and thereby increase survival. Basal levels as well as induced levels of nuclear p65 compared to the cytosolic pool following TNFa addition did not significantly differ from the parental line except for *Tnfrsf1a −/−* cells which showed as expected no response to TNFa (Fig. 5a). The transcriptional response as indicated by an NF-κB reporter assay was also unchanged (Fig. 5b). We then sought to determine if mutant cells could be re-sensitized to TNFa. Cycloheximide (CHX) is thought to sensitize cells to TNFa by translational inhibition of the short-lived c-FLIP (CFLAR) protein and subsequent activation of caspases, in particular Caspase-8. Following CHX treatment, all cell lines except *Tnfrsf1a −/−* were sensitive to TNFa with *Smg7 −/−* showing significantly less sensitivity than the parental cell line (Fig. 5c). Thus susceptibility to apoptotic challenge can be restored indicating that that the TNFa cell death pathway is not irreversibly blocked in *Smg7 −/− and Ces2a −/−* cells and that increased caspase activity can overcome this inhibition.

We also investigated the effect of TNFa treatment on core components of apoptosis in mutant cells. p53 (TRP53) is activated following DNA damage and SMG7 was recently reported to stabilize p53 following doxorubicin treatment [33]. We evaluated p53 levels in cells treated with TNFa and found that compared to the parental cell line *Smg7 −/−* cells showed a dramatic increase in p53 levels (Fig. 5d). *Ces2a −/−* cells also showed an increase in basal p53 levels that did not increase following TNFa treatment, while *Tnfrsf1a −/−* cells showed a decrease in basal p53 levels. Since elevated p53 is associated with cell death and Smg7 is reportedly associated with p53 in the cytoplasm we investigated if p53 localization may be affected in mutant cells. Following TNFa induction, the fraction of p53 in the nucleus compared to the cytoplasm increases significantly in control cells, whereas this ratio was constant in *Smg7 −/−* cells (Fig. 5e, f). Together these results show that p53 levels are increased in *Ces2a −/−* and *Smg7 −/−* cells and in the latter that p53 is decreased in the nucleus compared to controls.

### Further applications

ENCoRE was also tested for processing publicly available data. Bassett and colleagues [21] performed a CRISPR viability screen in Drosophila S2R+ cells and reported a lack of enrichment of essential genes in their dataset. Upon re-examination with ENCoRE, at least two highly scoring genes emerged that implicate cell cycle control (Z600, FANCI, see [Additional file 6] for volcano plots) in the enriched fraction, suggesting cell cycle dysregulation in the resulting mutant cells and accumulation of the associated sgRNAs. Other enriched or depleted guides have minimal overlap with the reported genes and could be retested on a candidate basis for viability impact. The differences in the analysis results of ENCoRE and DESeq2 [34] used by Bassett et al. may be due to different methods and filtering options applied to the data. DESeq2 was used to identify statistically significant changes in sgRNA counts for genes ≥3 sgRNAs based on a negative binomial distribution of the mean sgRNA counts. In contrast, ENCoRE performs a median imputation strategy with a subsequent z-test. The resulting ordered *p*-values correspond to a conservative ranking from a paired t-test. Especially the usage of the mean of sgRNA counts per gene can be problematic if outliers are present that cause artificial low or high mean values.

In addition, we tested the open-source software MAGeCK [22] and compared its negatively and positively selected genes from a CRISPR/Cas9 screening on mouse embryonic stem cells versus plasmid conditions with the analysis results of ENCoRE in [Additional file 6]. After median-normalization and estimation of the variance of the read counts MAGeCK tests whether sgRNAs differ significantly between treatment and control assuming a negative binomial distribution. Then MAGeCK ranks sgRNAs based on the calculated p-values and uses a modified robust ranking aggregation (RRA) algorithm afterwards. Although different methods are employed, ENCoRE identifies the same significantly negatively selected ribosomal genes *Rps5, GTF2B, KIF18B* and *Rpl19*. For positively selected genes *Zfp945* was identified by both programs, however ENCoRE and MAGeCK differed on their identification of *Trp53* which was found to be significant only by the latter in [Additional file 6, with step 5 of the MAGeCK documentation webpage]. We attribute the difference in these selected genes to the more stringent filtering performed by the ENCoRE FFM, which used a specific search string to identify guide sequences.

In summary, comparison of analyses of publicly available datasets and established software tools demonstrates that ENCoRE is able to identify essential genes from genome-scale CRISPR/Cas9 knockout screens by a relatively simple statistical model. In contrast to command line based programs like MAGeCK or web tools based on Z-scores such as ATARIS [35], ENCoRE serves up a simple and intuitive graphical user interface designed concisely for CRISPR screens. In addition, the processing of primary sequencing data is included in the same package and is very accurate due to the search function to identify search strings (e.g., barcodes) leading to a compact process throughput.

## Discussion

Technologies such as CRISPR/Cas9 based mutagenesis in somatic cells enable rapid workflows and large scale data generation that require comparably streamlined informatics. Here, our goal was to simplify the informatic workflow of scaled projects by creating a user-friendly GUI for next generation sequencing data processing as well as display an immediate assessment of results. In recessive screens, CRISPR/Cas9 sgRNA libraries are often directed to coding sequences and are constructed to have several guide target sequences per gene in order to guarantee mutagenicity. In most cases, simple selection with selective media will provide the most pure populations from which sequences can be derived. However, suboptimal selection conditions can also be used if subsequent informatics steps can yield relevant gene data. ENCoRE accomplishes this by processing sgRNA sequences from large datasets, filtering, and matching them to their respective gene sequences. It also uses a median imputation statistical method to compensate for missing or underrepresented guides. This method can, however, also produce artificially high or low values if one or two guides give a substantial increase and several imputed values that elevate a genes significance. However, in these cases as well median imputation is a valid strategy to compensate for discrepant guide sequences that can be quickly fine-tuned by manual curation.

The power of ENCoRE lies in its ability to rapidly distill large datasets into workable files and to give an overview of genes involved in a process. By virtue of its compact size and multithreaded processing, a typical desktop user can expect to rapidly filter and display results for large projects within a few hours. Thus comprehensive CRISPR/Cas9 screens and data analysis can be easily managed in laboratories without specialized informatics or cloud processing. ENCoRE is designed to be easily expandable with other functional panels by its modular architecture. The software is published and released as open source under the GNU license, so that users with java knowledge can implement their own modules for processing and reporting.

### Novel regulators of extrinsic apoptosis

In order to test if ENCoRE could rapidly deduce meaningful candidates from a novel dataset we conducted a recessive lentiviral sgRNA screen for genes that could give resistance to TNFa. Screens have been conducted before with RNA interference, however, major players were not identified [36] supporting the prowess of CRISPR to extensively map pathways. In our screen, stereotypical extrinsic apoptosis pathway members including *Tnfrsf1a*, *Casp8*, *Bid* and *Tnfrsf1b* were identified. Among the other new protein-coding genes identified we found *Smg7* and *Ces2a*, which when independently mutated strongly protected against TNFa-induced apoptotic cell death.

SMG7 acts in a complex of other factors including UPF1 to promote nonsense mediated decay (NMD) of RNA transcripts containing premature termination codons that cause protein truncations [37]. Currently it is unknown as to whether NMD is required to promote apoptosis or is simply tolerated as caspases appear to inhibit NMD during apoptosis [38]. Interestingly, alternative splicing has been determined to be one of the factors closely associated with NMD [37]. HNRNPF was also identified with high confidence as an apoptosis regulator in our screen and is known to play a role in RNA splicing, particularly of *Bcl-x* [39]. *Bcl-x* transcripts are spliced to either make an anti-apoptotic transcript *Bcl-xL* or a pro-apoptotic transcript *Bcl-xS*, and HNRNPF has been shown to aid in the production of the latter [28]. Therefore, it is possible that SMG7 could also participate in the stability of RNA isoforms by altering their ratios and promoting pro-apoptotic transcripts.

SMG7 (also known as Breast Cancer-Associated Antigen SGA-56 M) has been associated with cancer and is strikingly absent from a panel of 11 of 12 lymphoma samples tested for SMG7 antibody staining [40] suggesting that, as shown in our experiments, cells are resistant to apoptosis stimuli. Using individual cell lines containing mutations in both gene copies we show that *Smg7 −/−* cells are resistant to TNFa challenge and that cell death is caspase-dependent as demonstrated by zVAD protection (Fig. 4). We also performed tests in Smg7 mutant cells with known apoptosis-inducing chemotherapy drugs used in oncology. Whereas *Tnfrsf1a* mutant cells were partially resistant against Doxorubicin, *Smg7 −/−* cells showed only weak protection against this and other intrinsic inducers of apoptosis, demonstrating that global apoptosis is not inhibited per se. This is consistent with another report showing that *Smg7* mutation does not increase survival against doxorubicin, but rather counterintuitively, transiently increases the fraction of early apoptotic cells [33]. However, *Smg7 −/−* cells showed resistance to Erastin at lower concentrations, suggesting that Erastin-induced ferroptotic cell death is multimodal and may be potentiated by the TNFa pathway. Both *Smg7 −/−* and *Ces2a −/−* cells were weakly resistant to staurosporine, however, staurosporine is known to trigger an TNFa autocrine extrinsic pathway in certain cells [41]. Thus, *Smg7* is not exclusive to the extrinsic apoptosis pathway but it does appear to have a primary role in the promotion of TNFa-induced cell death. In contrast, mutation of *Ces2a* only showed robust protection against TNFa challenge, suggesting it is more specific to the extrinsic apoptosis pathway.

*Smg7 −/−* and *Ces2a −/−* cells show a normal NF-κB response following TNFa challenge (Fig. 5a,b). This also demonstrates that they do not act to restrict the pool of available TNFRSF1A receptor as seen in *Tnfrsf1a* mutant cells. Other extracellular ligands such as TNFSF10 (TRAIL) and FASL were inactive on all cell lines (data not shown) thus broad suppression of extrinsic cell death activity could not be tested. However, sensitivity to TNFa could be restored in mutant *Smg7 −/−* and *Ces2a −/−* cells by addition of cycloheximide, suggesting that the absence of these proteins can be overcome by activating caspases in the TNFa signaling pathway.

Mutation of *Smg7* results in a slight increase in p53 protein levels that increase dramatically following TNFa treatment (Fig. 5d). This is a surprising result, as p53 upregulation is typically associated with apoptosis and it is not known to be stabilized/upregulated following TNFa treatment. One possibility to explain this observation is that p53 cell death promoting activity may be compromised and its levels upregulated as a compensatory mechanism. We investigated whether p53 localization was affected and found that in control cells following TNFa treatment a slight but significant shift from cytosol to nucleus is seen, while in *Smg7 −/−* cells no such change is observed. Given that *Smg7 −/−* cells are partially resistant to Erastin/ferroptosis and that p53 is known to downregulate *SLC7A11* [42], a protective component of ferroptosis signalling, these results cumulatively suggest that SMG7 acts to facilitate p53 and that in its absence, the amplification of cell death signal is abrogated [43, 44], leading to cell survival.

Mutation of *Ces2a*, a carboxylesterase, was shown to be protective against TNFa-induced apoptosis in our screen and mutant cell line. Carboxylesterases are known to regulate hydrolyze a wide spectrum of xenobiotic and fatty acid esters and release them from cytoplasmic lipid compartments. Human CES2 also has the ability to catalyse the hydrolysis of retinyl palmitate to retinol [45, 46] and retinoic acid has been found to induce the expression of TNF receptors, TRAIL and caspase-8 in lung cancer cells resulting in acceleration of TNFa-induced apoptosis [47]. Thus, a plausible explanation for the discovery of *Ces2a* in a CRISPR screen for genes protecting against TNFa might be the reduced expression of pro-death factors downstream of vitamin A or its metabolites. We investigated the levels of p53 in *Ces2a −/−* cells and found surprisingly also an increase in basal levels, suggesting as in *Smg7 −/−* cells, p53 is associated with protection. However, its human orthologue CES2 appears to differ from its closely related neighbors CES1 and CES3 in substrate specificity [48] and could conceivably produce an additional pro-apoptotic metabolite as a consequence of its enzymatic activity.

## Conclusions

In summary, ENCoRE software enables bench scientists to rapidly distill CRISPR screening results into candidate gene lists, which can be further investigated in hypothesis-driven experiments. This is a critical improvement that encourages revisiting well-characterized pathways such as extrinsic apoptosis, as the discovery of *Smg7* and *Ces2a* confirm. By streamlining discovery informatics, the focus will naturally shift to delineate mechanism of action and the expansion our understanding of current pathway networks.

## Availability and requirements

Project name: ENCoRE (Easy NGS to CRISPR Results).

Project home page: http://www.helmholtz-muenchen.de/encore

Operating system(s): Platform independent.

Programming language: Java.

Other requirements: Java Runtime Environment (JRE) 8 (64 bit), 4 GB RAM and 2 CPUs.

License: GNU GPLv3.

## Additional files

Additional file 1: A list of primer sequences used in the study. (XLS 35 kb)

Additional file 2: The ENCoRE Quick Guide for downloading and processing CRISPR screens with ENCoRE. (PDF 942 kb)

Additional file 3: Venn diagrams showing overlap of top 20 genes identified by different software ENCoRE, MAGeCK, and edgeR for two additional datasets, (A) Koike-Yusa, et al., [4] and (B) Bassett, et al., [21]. For both datasets, a comparable overlap of top genes is seen between pairs ENCoRE/MAGeCK and ENCoRE/edgeR as observed between MAGeCK/edgeR. (PDF 227 kb)

Additional file 4: TNFa kill curve for CRISPR/Cas9 screening. Concentrations of soluble TNFa compared to cell survival 24 h after addition to murine fibroblasts. Addition of the pan-caspase inhibitor zVAD demonstrates caspase-dependent cell death. (PDF 6 kb)

Additional file 5: Genotyping data for mutant cell lines. Individually generated mutations in mouse fibroblasts were amplified from genomic DNA, cloned, sequenced, and characterized for indels. For each gene primary sequencing data from the parental cell line (wild-type) are aligned with mutated sequences (KO) resulting in a frameshift mutation. The CRISPR guide sequences for engineering the mutations are also aligned (red). (PDF 949 kb)

Additional file 6: ENCoRE validation on publicly available CRISPR screening data. (A) ENCoRE output from Bassett and colleagues [21] CRISPR viability screen in Drosophila S2R+ cells. At least two highly scoring genes emerged that implicate cell cycle control (Z600, FANCI) not seen in the original publication. (B) ENCoRE output from a CRISPR/Cas9 screen on mouse ESC cells from Li and colleagues [22] shows a similar profile of negatively selected genes and ZFP945 for positively selected genes, but differs in the identification of TRP53. (PDF 494 kb)

## Abbreviations

5-FU: 5-Fluorouracil
*Ces2a*: Carboxylesterase 2a
CFLAR: c-FLIP
CHX: Cycloheximide
CRM: CRISPR report module
CSV: Comma separated values
DCA: Deoxycholic acid
ENCoRE: Easy NGS-to-Gene CRISPR REsults
FFM: FASTQ filter module
GUI: Graphical user interface
*Hnrnpf*: Heterogeneous nuclear ribonucleoprotein F protein
indel: Insertions or deletions
NGS: Next generation sequencing
NHEJ: Non-homologous end-joining
NMD: Nonsense mediated decay
RRA: Robust ranking aggregation
sgRNA: Single guide RNA
*Smg7*: Suppressor with morphological defects in genitalia 7
TNFa: Tumor Necrosis Factor-alpha
TRAIL: TNFSF10
TRP53: p53
zVAD: z-VAD-FMK
