# Supplementary material for: ENCoRE: an efficient software for CRISPR screens identifies new players in extrinsic apoptosis
Source: BMC Genomics. 2017 Nov 25;18:905. doi: 10.1186/s12864-017-4285-2 (PMC5702081; doi:10.1186/s12864-017-4285-2)
Supplement: Supplementary file 2 — The ENCoRE Quick Guide for downloading and processing CRISPR screens with ENCoRE. (PDF 942 kb) [file 12864_2017_4285_MOESM2_ESM.pdf]

ENCoRE: Easy NGS to CRISPR Results

Quick Guide

1 Contents

1 Contents 1

2 System requirements 2

3 Menu 2

4 Encore Process Overview 2

5 FASTQ Filter 3

5.1 Overview 3

5.2 Workflow & Preview 4

5.3 Variable Filters 4

5.3.1 Quality 4

5.3.2 Misreads 5

5.3.3 Length 5

5.3.4 Delete 6

5.3.5 Crop 7

5.3.6 Replace 7

5.3.7 Search 8

6 CRISPR Report 9

6.1 Overview 9

6.2 Basic Information 10

6.3 Statistics Panel 10

6.4 CSV output file 12

6.5 The ENCoRE Java Project 12

7 Appendix 13

2 System requirements

- JAVA Runtime environment (version 8, 64 bit), 4 GB RAM and two CPUs
- For FASTQ files with more than 50 million reads, > 4 GB and > 4 CPUs

It is recommended that all files are opened from a local drive. Opening files from a network resource or an external device (e.g., USB stick) may cause the program to stall.
ENCoRE can be downloaded from: http://www.helmholtz-muenchen.de/encore

3 Menu

Via the menu one can: open new modules in additional tabs, exit the program or access general information in the „About“ dialog. There are two modules available:

1. FASTQ Filter: manipulate FASTQ files
2. CRISPR Report: evaluate filtered FASTQ files based on CRISPR screening

The modules are explained in the following sections. An example section contains a guide through a standard evaluation procedure.

4 Encore Process Overview

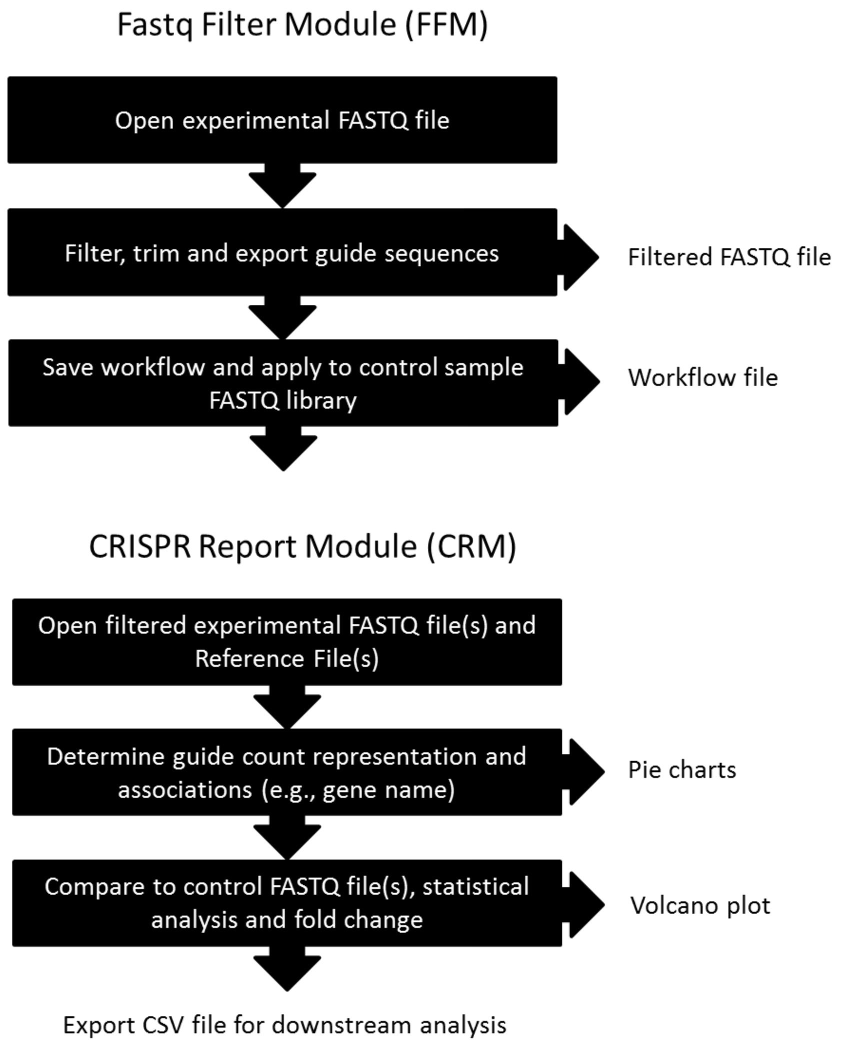

5 FASTQ Filter

5.1 Overview

The FASTQ Filter module enables one to alter raw FASTQ files. For that purpose one can build or import a sequential workflow of different filters that will be applied to datasets. Together with an instant preview, this powerful module makes it easy to adjust FASTQ files to individual needs.

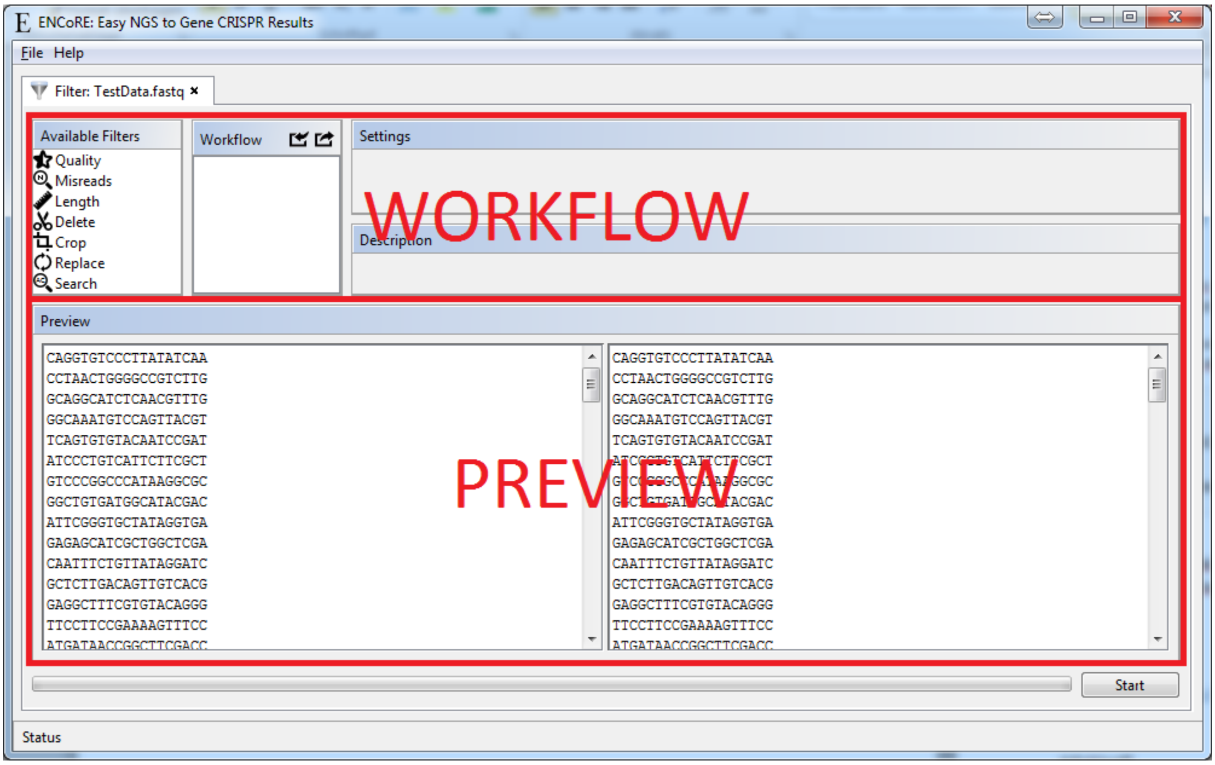

To open a FASTQ file for processing, click File→Open→FASTQ Filter. Choose a FASTQ file in the dialog appearing and click „OK“. This will open a new tab showing different panels containing a workflow on the top and a preview panel on the bottom.

Note: The FASTQ file must be in the following format. Any corrupt data within the file will be ignored. For more information see https://en.wikipedia.org/wiki/FASTQ_format.

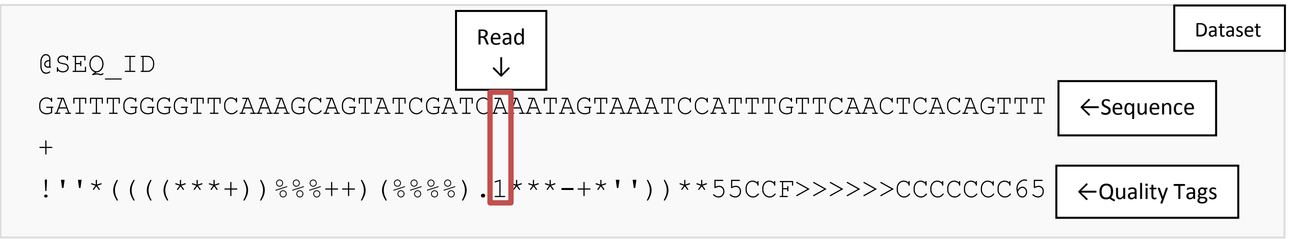

5.2 Workflow & Preview

In the workflow section one can build a workflow of manipulations to make on a FASTQ file.

To add a filter to a workflow, double-click the filter or drag-and-drop the filter from the „*Available Filters*“ panel to the „Workflow“ panel. The addition of a filter will allow the initial preview of the sequences and appearance after filtering. From this point, it is also possible to export/import an existing workflow via the buttons on the top right corner of the „*Workflow*“ panel.

Once several filters are added to the workflow they can be rearranged by drag-and-drop. To rearrange multiple filters at the same time, one can mark filters with „shift“ or „control“ pressed while clicking on the filters. For deleting filter(s), mark them and press “delete”.

If one clicks on a workflow filter, the corresponding settings and descriptions of this filter are shown. For a detailed description about filters and settings see 5.3.

Every time the settings are changed, the preview will immediately be updated. For this update, all filters, from the first to the last selected filter, are sequentially applied to the datasets. The original file will not be altered.

After building a workflow one can start the processing of a FASTQ file by pressing the start button. A file dialog will appear where one can select where to save the filtered FASTQ file. The processing can take a few seconds up to several minutes, depending how large the original file is and how many filters were applied. The bar at the bottom shows the progress.

ATTENTION: the datasets of the filtered file will not be in the same order as the original file due to the nature of parallel processing!

5.3 Variable Filters

The filters available should cover all possible manipulations on a FASTQ file. There are also more sophisticated filters, like „replace“ and „search“ available. Here is a detailed description of all filters, how they work and further information about the settings.

5.3.1 Quality

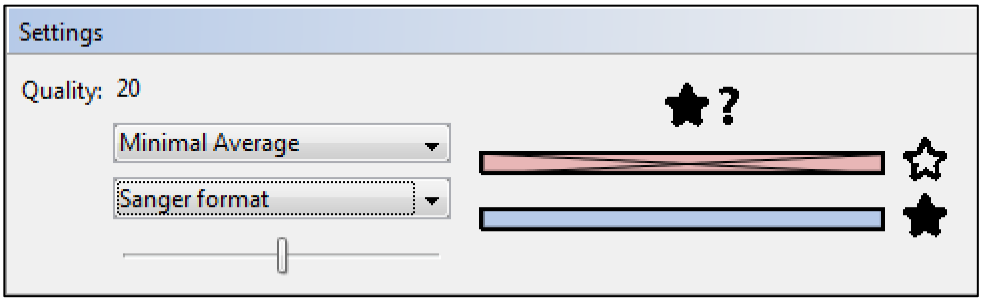

The Quality filter is applied to filter sequences by different quality criteria. All FASTQ files come in formats determined by the NGS service facility. The formats have different quality ranges. Choose a format and adjust the slider to a desired quality threshold.

ATTENTION: If applying more than one filter, quality should be performed first due to loss of quality data in subsequent filtering steps.

The following criteria/constraints can be applied:

- **Minimal Average**: Averages the quality of all single reads in the dataset. If the average is *below* the defined quality score the whole dataset will be discarded.
- **Maximal Average**: Averages the quality of all single reads in the dataset. If the average is *above* the defined quality score the whole dataset will be discarded.
- **Lower Threshold**: If the quality of only one read is *below* the quality score, the whole dataset will be discarded.
- **Upper Threshold**: If the quality of only one read is *above* the quality score, the whole dataset will be discarded.

5.3.2 Misreads

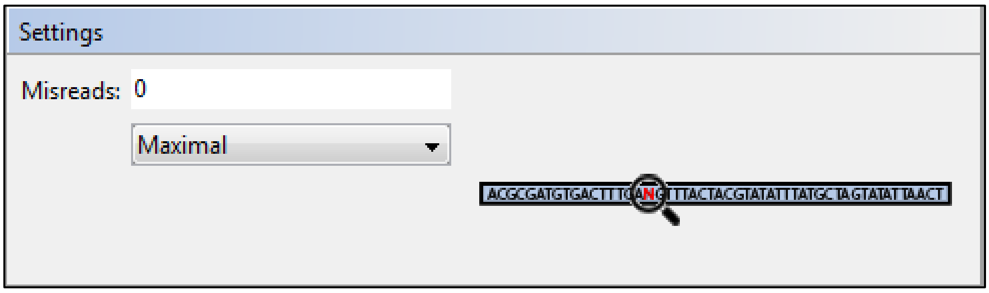

The Misreads filter is applied to filter datasets based on the number of misreads. Depending on the reading mechanism of the NGS service there may be misreads (marked with a sequence tag „**N**“) in the dataset. To filter such datasets, one can determine an amount of misreads together with a filter criteria.

Following criteria/constraints can be applied:

- **Maximal**: If the sequence contains *more* misreads than the defined number, the entire dataset will be discarded.
- **Minimal**: If the sequence contains *less* misreads than the defined number, the entire dataset will be discarded.
- **Exact**: If the sequence contains *more or less* misreads than the defined number, the entire dataset will be discarded.

5.3.3 Length

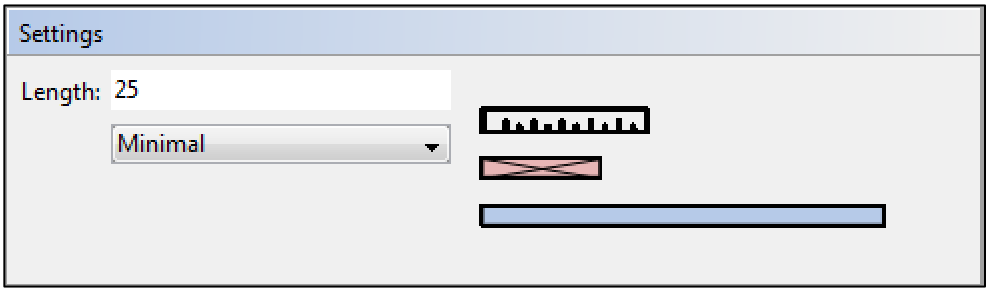

The Length filter is applied to filter datasets based on their length. To filter such datasets one can determine a numerical length together with filter criteria.

Following criteria/constraints can be applied:

- **Minimal**: If the sequence is *shorter* than the defined number, the whole dataset will be discarded.
- **Maximal**: If the sequence is *longer* than the defined number, the whole dataset will be discarded.
- **Exact**: If the sequence is *shorter or longer* than the defined number, the whole dataset will be discarded.

5.3.4 Delete

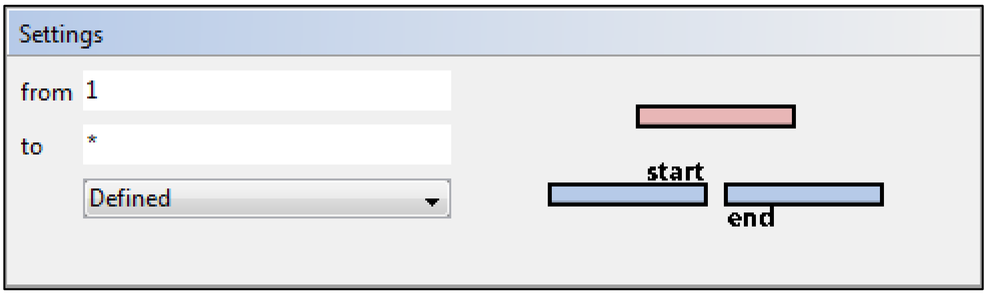

The Delete filter is applied to alter the length of datasets.

Following criteria/constraints can be applied:

- **Defined**: Deletes a defined range (from → to) of reads from the dataset. The dataset will be discarded if:
  - The from value is less than the to value
  - The range of reads to be deleted is bigger than the total length of the sequence
  - The to value exceeds the length of the sequence

  ATTENTION: If one wishes to delete reads from a given *from* value to the end of the sequence, use an asterisk (*****) as a *to* value.
- **Front**: Deletes the defined number of reads from the *beginning* of the sequence. If the sequence of a dataset is shorter than the defined number, the complete dataset will be discarded.
- **Back**: Deletes the defined number of reads from the *end* of the sequence. If the sequence of a dataset is shorted than the defined number, the complete dataset will be discarded.

5.3.5 Crop

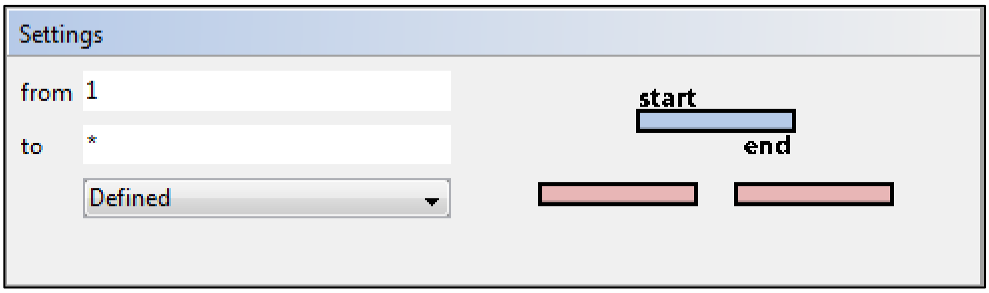

The Crop filter is applied to only take a subset of sequences of the dataset.

Following criteria/constraints can be applied:

- **Defined**: Takes a defined range (from → to) of reads from the dataset and discards the remaining. The dataset will be discarded if:
  - The from value is less than the to value
  - The to value exceeds the length of the sequence

  ATTENTION: If one wishes to take reads from a given *from* value to the end of the sequence, use an asterisk (*****) as a *to* value.
- **Front**: Takes the defined number of reads from the *beginning* of the sequence. If the sequence of a dataset is shorter than the defined number, the complete dataset will be discarded.
- **Back**: Takes the defined number of reads from the *end* of the sequence. If the sequence of a dataset is shorted than the defined number, the complete dataset will be discarded.

5.3.6 Replace

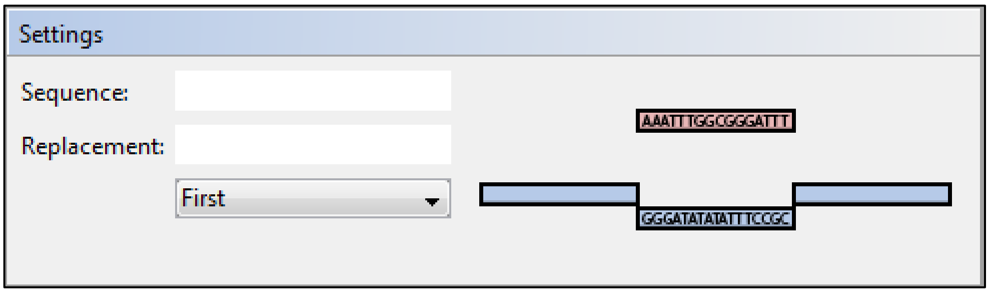

The Replace filter is applied to replace a subset of sequences in the dataset with another. Define a *Sequence* to search for and a *Replacement* sequence together with a constraint. The *Replacement* sequence can be empty, if one wishes to delete a specific sequence. A replacement will only happen if the given sequence will be found. Please note that the quality tags of the replacement will be the lowest quality tags and will therefor alter the quality of the complete dataset negatively!

Following criteria/constraints can be applied:

- **All**: All found sequences will be replaced.

- **First**: Only the first iteration of the sequence will be replaced.
- **Beginning**: Replaces the beginning of the sequence.
- **End**: Replaces the end of the sequence.

CAUTION: This filter may negatively alter quality tags! Do not attempt to chain a quality filter afterwards!

5.3.7 Search

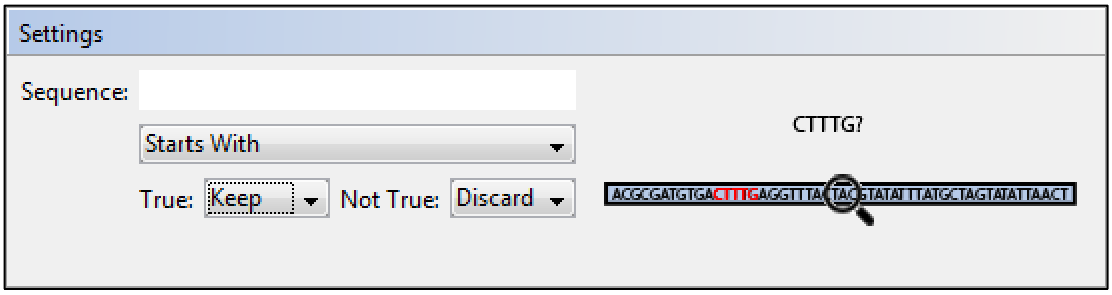

The Search filter is applied for a more sophisticated manipulation of a dataset. Here a sequence should be defined to search for together with a constraint. Then what should happen if the sequence is found and meets the constraint („*True*“) or not („*Not True*“) should be defined.

Following criteria/constraints can be applied:

- **Starts with**: The dataset sequence must *start* with the search sequence.
- **Ends with**: The dataset sequence must *end* with the search sequence.
- **Contains:** The dataset sequence must *contain* the search sequence.
- **Discard:** Discard the dataset.
- **Keep:** Keep the dataset.
- **Crop:** Crops subsets of the dataset sequence depending on the following additional constraints:
    - First: Crops the first found sequence from the dataset and discards the remaining.
    - Before: Keeps everything before the first found sequence.
    - Before Including: Keeps everything before the first found sequence, including the found sequence.
    - After: Keeps everything after the first found sequence.
    - After Including: Keeps the first found sequence and everything after.
- **Delete:** Deletes subsets of the dataset sequence depending on the following additional constraints:
    - First: Deletes the first found sequence from the dataset.
    - Before: Deletes everything before the first found sequence.
    - Before Including: Deletes everything before the first found sequence, including the found sequence.
    - After: Deletes everything after the first found sequence.
    - After Including: Deletes the first found sequence and everything after.

CAUTION: This filter may negatively alter quality tags! Do not attempt to chain a quality filter afterwards!

6 CRISPR Report

6.1 Overview

The CRISPR Report module is designed to evaluate and provide statistics for filtered FASTQ files from a CRISPR screening experiment. This module counts guide sequences in a FASTQ file and labels them (with gene names, for example) with the use of a CRISPR reference file. Subsequently, the module is able to compare an experimental FASTQ file with a control library to determine guide count changes in the experiment.

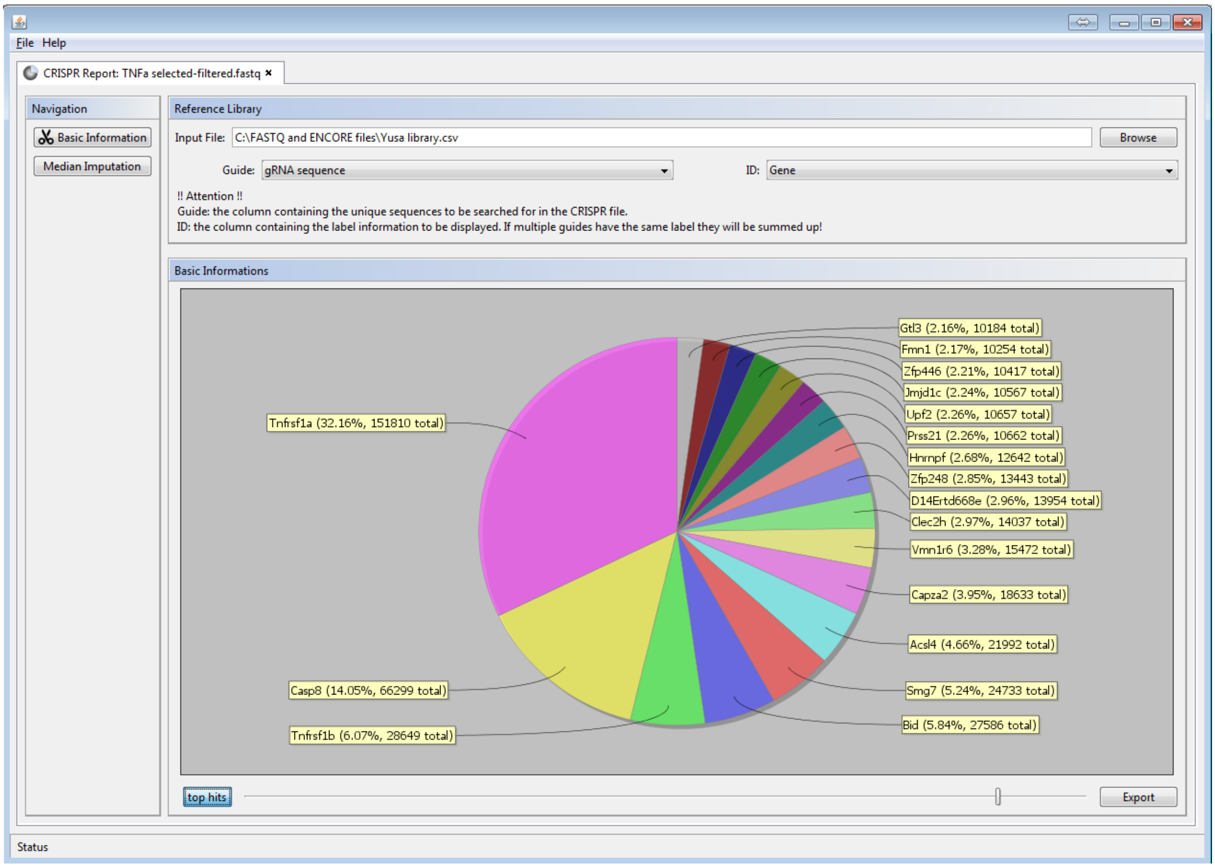

To open a new CRISPR report panel click File→Open→CRISPR Report. Choose an experimental FASTQ file containing filtered and trimmed guide sequences in the dialog appearing and click „OK". This will import the FASTQ file. Please note that this process can take a few seconds, depending on CPU power.

The FASTQ file should contain filtered and trimmed sequences guide sequences one wishes to cross-reference with a CRISPR reference file to get, for example, gene names. During the import process datasets with the same sequences are aggregated and counted. The final imported data does not contain any duplicates.

CAUTION: Guide sequences should be trimmed to match exactly to sequences in the reference file (e.g., with or without adjacent PAM sequences).

6.2 Basic Information

The Basic Information Panel is used to label the counts of unique sequences from the imported FASTQ file. To display a pie chart of the data one needs to add a reference file by clicking on the „Browse“ button. **IMPORTANT**: The reference file must be in .CSV format and containing headers. After the library is imported one can select a „Guide“ sequence to match to and an „ID“ that should be displayed in the chart. The headers found in the reference file are shown as drop-down lists.

The „Guide“ column should contain the filtered FASTQ derived sequences to be cross-referenced to the CRISPR reference file (e.g. "gRNA sequence").

CAUTION: Please make sure that all guides in the reference file are unique!

The „ID“ column should contain the alphanumeric string that will represent the label in the pie chart (e.g., “Gene”). If multiple guides have the same label they will be aggregated.

CAUTION: If the .CSV file contains the same label for different guides, all the guides will be aggregated! If unique names are wished, please adjust the reference file to contain a column with unique gene guide labels (e.g., Gene_x_1).

After one has selected the Guide and the ID, the pie chart will be populated with the corresponding data.

Below the chart is a slider to adjust the amount of the displayed top guides. If one wants to turn off the display of the remaining guides the shortcut „top hits“ button can be activated.

CAUTION: The displayed percentage with an activated „top hits“ button does not reflect the actual percentage of the whole dataset but just of the cohort shown.

The „Export“ button in the bottom right corner exports the guide count with the chosen ID label in .CSV format for further processing.

6.3 Statistics Panel

The Statistics Panel is used to compare filtered and trimmed experimental FASTQ files to (control) FASTQ datasets. It is assumed that all datasets were processed with the same workflow to give guide sequences with the same format. Please make sure that a reference file is already selected with the Guide/ID column within it (see 6.2). To compensate for artificially high, low, or missing guides a median imputation strategy is used (see manuscript text for details). In addition, a total normalization is performed to correct for differences between the libraries. Normalization is applied by dividing each median imputated count of reads for a given library through the sum of all median imputated values of this library.

Minimally, three files are necessary for calculating p-values and fold change:

1. The experimental FASTQ file(s), filtered and trimmed
2. The control FASTQ file(s), filtered and trimmed
3. The reference file (as CSV) containing guide sequences and corresponding information

Optionally, *n* experimental and control replicate FASTQ files can be entered. The application was tested for five experimental and five control libraries but many more should be possible. The only

restriction for the number of replicates is the size of the RAM storage capacity which is needed when the data matrix is constructed.

The median imputation strategy as well as total normalization and the computation of p-values and false discovery rates (FDRs) are extended to *n* replicates. To display the fold change chart one first selects a Control Library (FASTQ format) to compare to the experimental FASTQ dataset. After the import is done one should see a volcano plot:

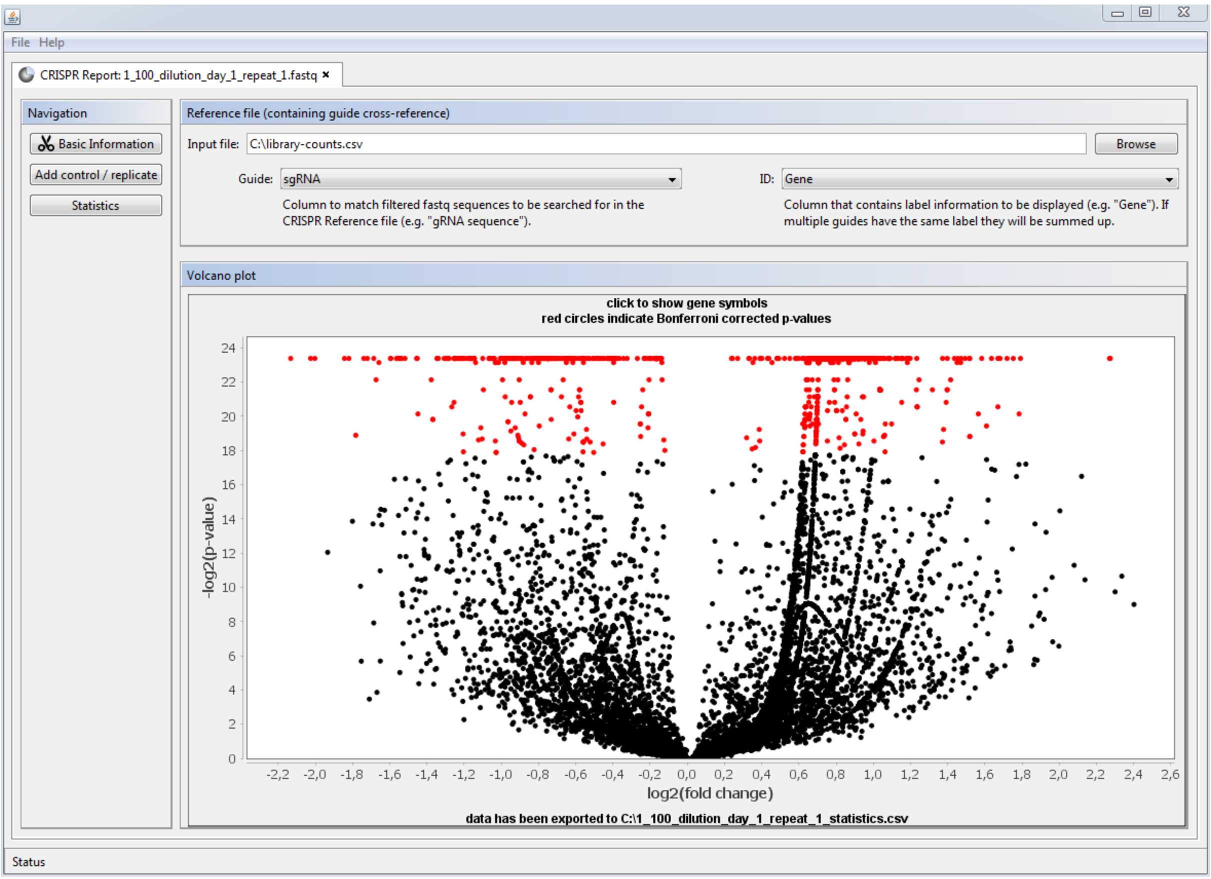

The x-axis represents the inverse $\log_2$(fold change) of the experimental values of the corresponding ID you have chosen in your reference file compared to the control values. „Accumulated" means that the fold changes are combined for every matched guide (see manuscript text for details).

The y-axis represents the inverse $\log_2$(p-value) of this ID compared to the control dataset. This only makes sense if the guides were accumulated because more than one guide exists for each ID, e.g. five guide sequences to one gene name.

To navigate the chart: mouseover shows the ID as well as the values for the x- and y-axes. A left click on the chart points makes the corresponding ID continually visible. A right click opens a context menu with further Options, like zooming or exporting the chart view.

6.4 CSV output file

The results of statistical analysis are written into a CSV formatted file and stored in the same directory from which the experimental FASTQ file was entered. The output file consists of the following columns:

- Gene symbol: derived from the reference file
- FDR(Bonferroni): corrected p-value according to the method of Bonferroni
- FDR(BH): corrected p-value after multiple testing by the method of Benjamini and Hochberg
- p-value: calculated by a normal z-test, corresponding to a paired t-test
- -log2(p-value): inverse log_2 based p-value
- log2(Fold change): log_2 based mean of the ratios of median imputated read counts from the sgRNAs in the experimental versus control library per gene
- #reads imputated control *1 to m* (replicate *1 to n*): median imputated read counts of *m* sgRNAs from *n* control libraries; missing read counts of sgRNAs per gene are set to one and subsequently counts of one are replaced by the median
- #reads imputated condition *1 to m* (replicate *1 to n*): median imputated read counts of *m* sgRNAs from *n* experimental libraries; missing read counts of sgRNAs per gene are set to one and subsequently counts of one are replaced by the median
- #reads control *1 to m* (replicate *1 to n*): "original" control read counts of the sgRNAs per gene; corresponding control/experimental read counts of a given sgRNA for a gene are set to one if one of both is missed
- #reads condition *1 to m* (replicate *1 to n*): "original" experimental read counts of the sgRNAs per gene; corresponding experimental/control read counts of a given sgRNA for a gene are set to one if one of both is missed

Columns referring to control and condition read counts "*1 to m*" imply arbitrary numbers assigned to a given sgRNA sequence. If more than one control and one experimental library are used then the arbitrary number of replicate files is shown in brackets.

Median imputated read counts for the control and experimental library/libraries from the CSV output file can be used to compute p-values based on a generalized linear model (GLM) which does not assume normal distribution or to calculate p-values from the distribution free method of a Wilcoxon signed-rank statistic with help of R statistical software or any other suitable statistic program.

6.5 The ENCoRE Java Project

The ENCoRE software tool is an open source, stand-alone Java program suitable for Windows, Macintosh and Linux operating systems. It can be redistributed and improved under the terms of the GNU licence GPLv3.

The GUI of ENCoRE was developed by using AWT and Swing Java libraries. Charts (e.g. the volcano plot) were realized with help of the JFreeChart Class Library. In order to accelerate the input of the input of FASTQ files packages from the MBassador project were used. MBassador represents a

feature-rich Java event bus optimized for high-throughput in multi-threaded environments. The Apache Commons Mathematics Library was applied to calculate the statistical result.

7 Appendix

Report any bugs to:

dietrich.truembach@helmholtz-muenchen.de
