# Supplementary figures and images for: ENCoRE: an efficient software for CRISPR screens identifies new players in extrinsic apoptosis
Source: BMC Genomics. 2017 Nov 25;18:905. doi: 10.1186/s12864-017-4285-2 (PMC5702081; doi:10.1186/s12864-017-4285-2)

Additional File 3

A

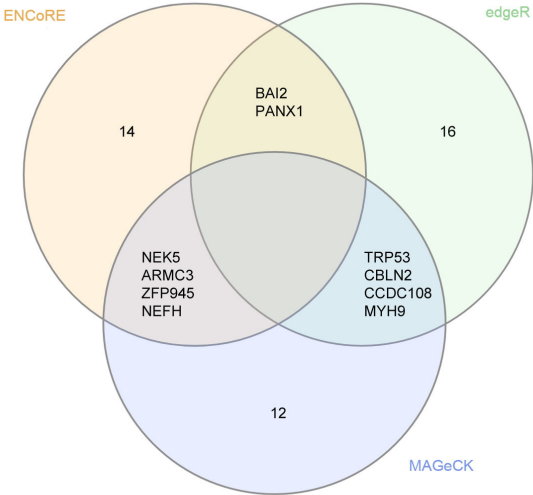

B

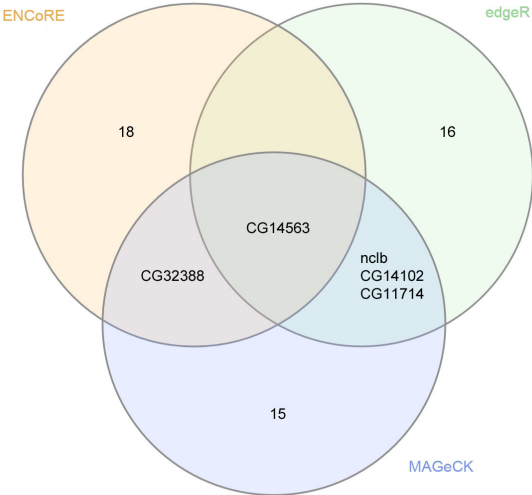

Supplement: Supplementary file 3 — Venn diagrams showing overlap of top 20 genes identified by different software ENCoRE, MAGeCK, and edgeR for two additional datasets, (A) Koike-Yusa, et al., [4] and (B) Bassett, et al., [21]. For both datasets, a comparable overlap of top genes is seen between pairs ENCoRE/MAGeCK and ENCoRE/edgeR as observed between MAGeCK/edgeR. (PDF 227 kb) [file 12864_2017_4285_MOESM3_ESM.pdf]

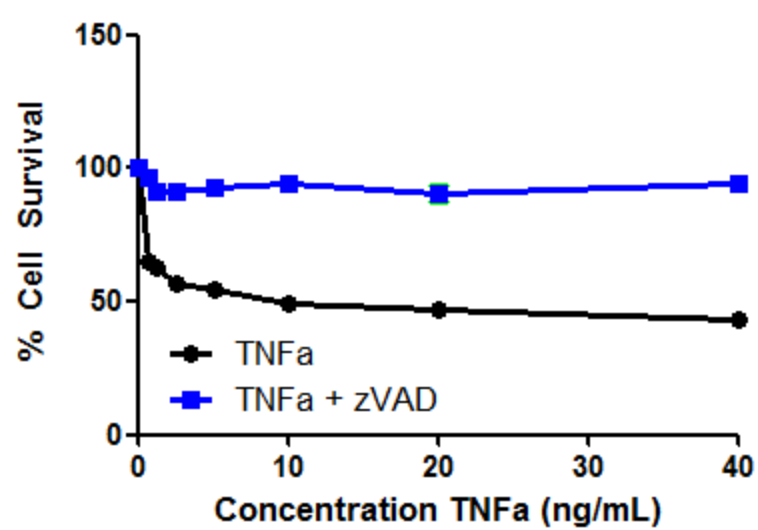

Supplement: Supplementary file 4 — TNFa kill curve for CRISPR/Cas9 screening. Concentrations of soluble TNFa compared to cell survival 24 h after addition to murine fibroblasts. Addition of the pan-caspase inhibitor zVAD demonstrates caspase-dependent cell death. (PDF 6 kb) [file 12864_2017_4285_MOESM4_ESM.pdf]

**A**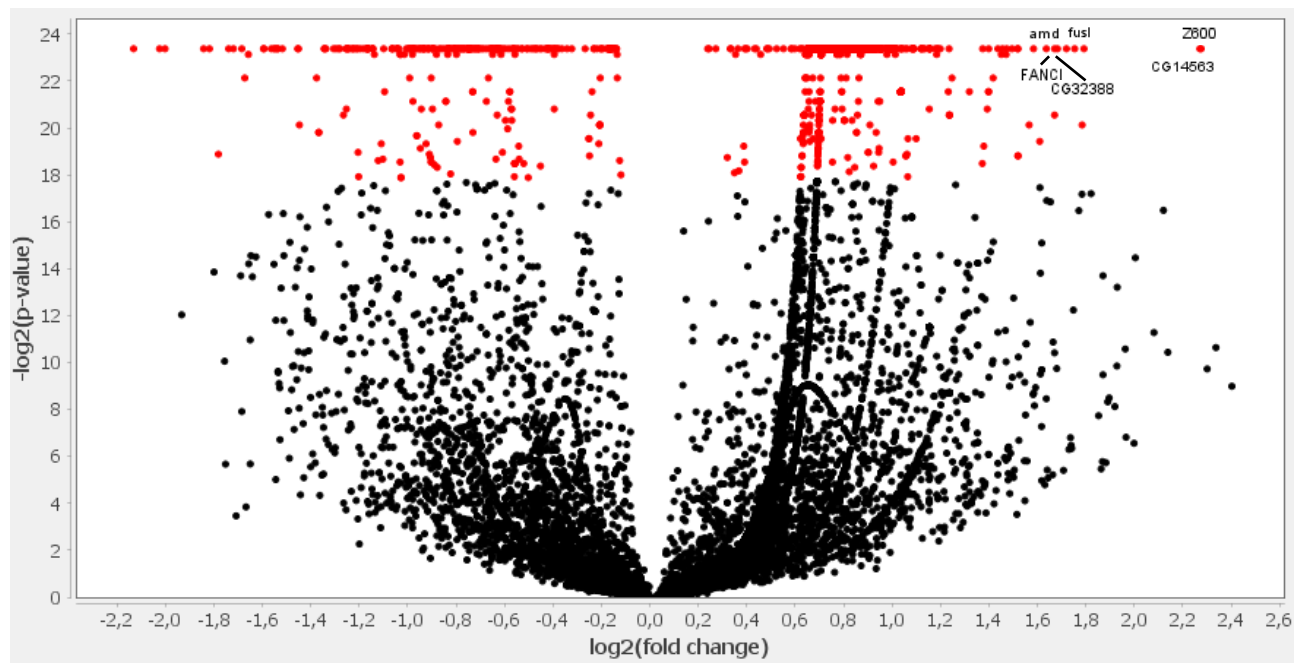**B**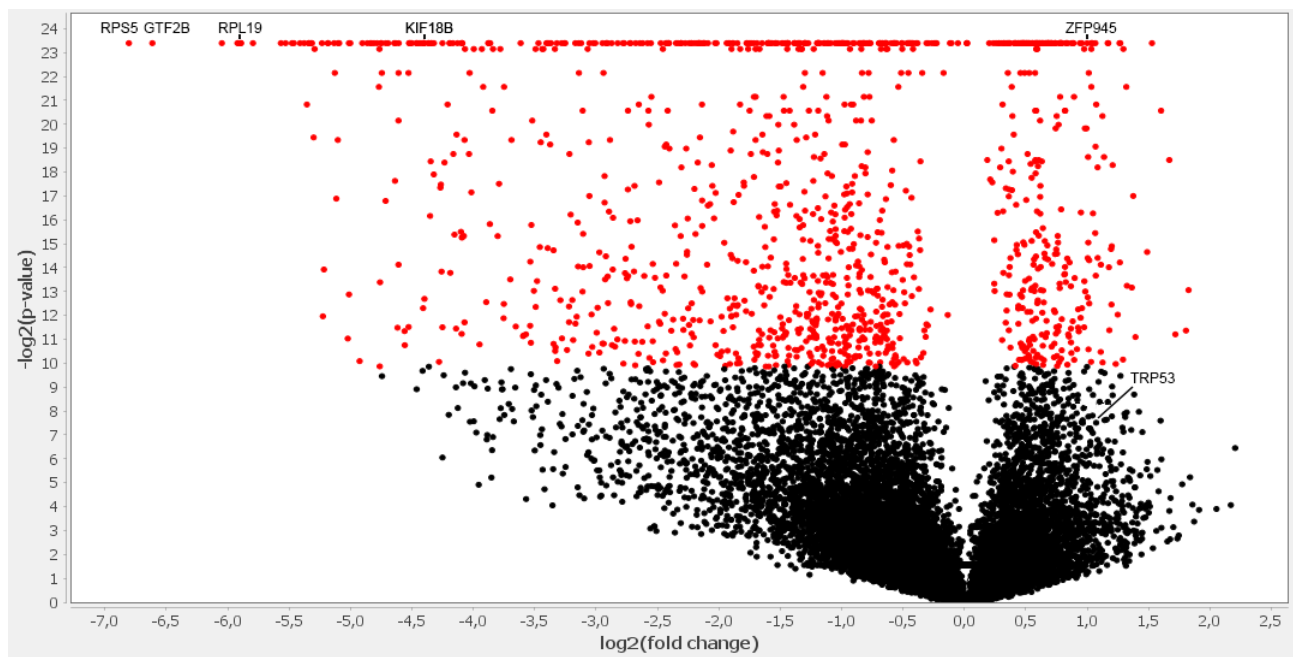

Supplement: Supplementary file 6 — ENCoRE validation on publicly available CRISPR screening data. (A) ENCoRE output from Bassett and colleagues [21] CRISPR viability screen in Drosophila S2R+ cells. At least two highly scoring genes emerged that implicate cell cycle control (Z600, FANCI) not seen in the original publication. (B) ENCoRE output from a CRISPR/Cas9 screen on mouse ESC cells from Li and colleagues [22] shows a similar profile of negatively selected genes and ZFP945 for positively selected genes, but differs in the identification of TRP53. (PDF 494 kb) [file 12864_2017_4285_MOESM6_ESM.pdf]
