# Supplementary material for: ENCoRE: an efficient software for CRISPR screens identifies new players in extrinsic apoptosis
Source: BMC Genomics. 2017 Nov 25;18:905. doi: 10.1186/s12864-017-4285-2 (PMC5702081; doi:10.1186/s12864-017-4285-2)

Additional File 5

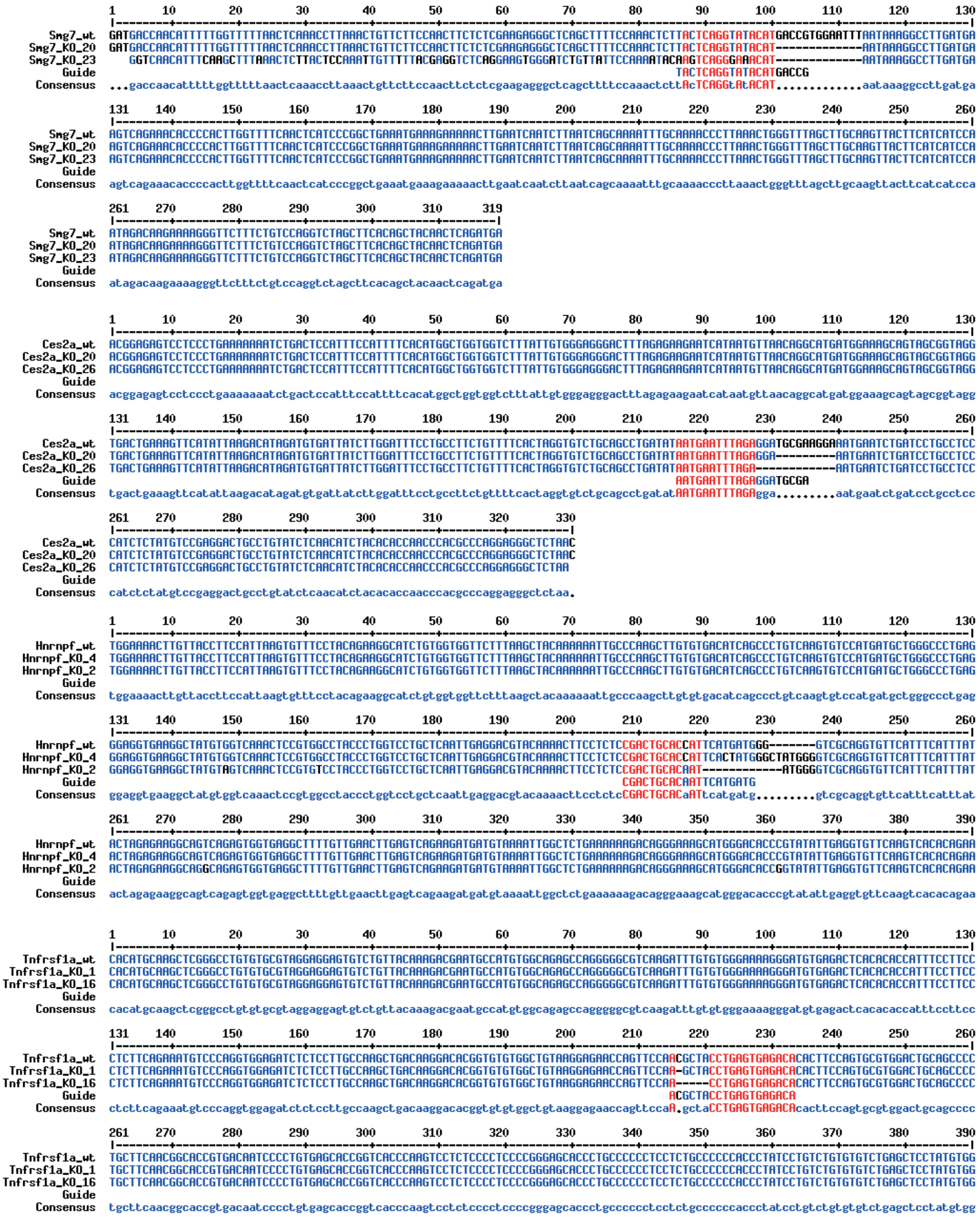

Supplement: Supplementary file 5 — Genotyping data for mutant cell lines. Individually generated mutations in mouse fibroblasts were amplified from genomic DNA, cloned, sequenced, and characterized for indels. For each gene primary sequencing data from the parental cell line (wild-type) are aligned with mutated sequences (KO) resulting in a frameshift mutation. The CRISPR guide sequences for engineering the mutations are also aligned (red). (PDF 949 kb) [file 12864_2017_4285_MOESM5_ESM.pdf]
